# Diabetes Engagement and Activation Platform for Implementation and Effectiveness of Automated Virtual Type 2 Diabetes Self-Management Education: Randomized Controlled Trial

**DOI:** 10.2196/26621

**Published:** 2021-03-29

**Authors:** Roy Sabo, Jo Robins, Stacy Lutz, Paulette Kashiri, Teresa Day, Benjamin Webel, Alex Krist

**Affiliations:** 1 Virginia Commonwealth University Richmond, VA United States; 2 Privia Health, LLC Arlington, VA United States

**Keywords:** type 2 diabetes mellitus, self-management education, patient engagement, informatics

## Abstract

**Background:**

Patients with type 2 diabetes require recommendations for self-management education and support.

**Objective:**

In this study, we aim to design the Diabetes Engagement and Activation Platform (DEAP)—an automated patient education tool integrated into primary care workflow—and examine its implementation and effectiveness.

**Methods:**

We invited patients aged 18-85 years with a hemoglobin A_1c_ (HbA_1c_) level ≥8 to participate in a randomized controlled trial comparing DEAP with usual care. DEAP modules addressing type 2 diabetes self-management education and support domains were programmed into patient portals, each with self-guided educational readings, videos, and questions. Care teams received patient summaries and were alerted to patients with low confidence or requesting additional help. HbA_1c_, BMI, and systolic and diastolic blood pressure (DBP) were measured.

**Results:**

Out of the 680 patients invited to participate, 337 (49.5%) agreed and were randomized. All of the 189 intervention patients accessed the first module, and 140 patients (74.1%) accessed all 9 modules. Postmodule knowledge and confidence scores were high. Only 18 patients requested additional help from the care team. BMI was lower for intervention patients than controls at 3 months (31.7 kg/m^2^ vs 32.1 kg/m^2^; *P*=.04) and 6 months (32.5 kg/m^2^ vs 33.0 kg/m^2^; *P*=.003); improvements were even greater for intervention patients completing at least one module. There were no differences in 3- or 6-month HbA_1c_ or blood pressure levels in the intent-to-treat analysis. However, intervention patients completing at least one module compared with controls had a better HbA_1c_ level (7.6% vs 8.2%; *P*=.03) and DBP (72.3 mm Hg vs 75.9 mm Hg; *P*=.01) at 3 months.

**Conclusions:**

The findings of this study concluded that a significant proportion of patients will participate in an automated virtual diabetes self-management program embedded into patient portals and health systems show promise in helping patients manage their diabetes, weight, and blood pressure.

**Trial Registration:**

ClinicalTrials.gov NCT02957721; https://clinicaltrials.gov/ct2/show/NCT02957721

## Introduction

### Background

Type 2 diabetes (T2D) affects an estimated 34 million people in the United States [1], costing US $327 billion annually [2]. T2D prevalence in the United States is expected to increase, whereas costs are expected to double over the next 25 years [3,4]. T2D self-management education and support (DSMES) provides individuals with the information and problem-solving skills needed to self-manage T2D and has been shown to improve medication adherence, self-blood glucose monitoring, glycemic control, and dietary behaviors [5,6] and reduce complications from uncontrolled T2D [7,8]. The American Diabetes Association (ADA) recommends the provision of DSMES for every patient at 4 points: at diagnosis, annually thereafter, when complicating factors arise, and when transitioning to new care teams [9].

Despite its proven effectiveness, many patients do not receive DSMES. Of the patients referred, only 23%-66% follow through to receive DSMES [10] because of barriers such as time commitments, schedule conflicts, or transportation difficulties [7]. Innovative DSMES delivery methods are needed to better meet patients’ needs and leverage limited resources.

Health information technology, specifically personal health records (PHRs) integrated into electronic health records (EHRs), has the potential to increase patient access to DSMES by automating the provision of educational content and allowing patients to review and complete programs at convenient times and locations [11]. Integrated PHRs can help automate identifying patients needing additional help, allow patients to initiate requests for support, and alert team members to initiate care or direct patients to existing community resources [12,13].

### Objectives

To help leverage the benefits of health information technology in providing DSMES, we created the Diabetes Engagement and Activation Platform (DEAP), which is an automated patient educational tool integrated directly into the primary care workflow. DEAP is accessed from the patient portal, consists of 9 modules that address the recommended ADA domains of diabetes education, assesses patients’ knowledge and confidence in managing each domain, and alerts care team members of patient needs. We aim to conduct a randomized controlled trial (RCT) to evaluate the implementation of DEAP and its effectiveness relative to usual care for improving patient T2D outcomes.

## Methods

### Overview

We conducted a patient-level RCT evaluating the implementation and effectiveness of DEAP with respect to changes in glycated hemoglobin (HbA_1c_; primary outcome), BMI, and blood pressure (BP) from baseline to 3 and 6 months. The study was conducted between November 1, 2017, and May 7, 2018, to achieve 6 months of patient tracking. This study was approved by the Virginia Commonwealth University Institutional Review Board and registered at ClinicalTrials.gov (identifier NCT02957721).

### Setting

A total of 21 practices spanning 5 states from the Privia Health, LLC (Privia), a technology-enabled, physician enablement company that collaborates with medical groups, health plans, and health systems, were recruited to participate in this study. The practices predominantly serve commercially insured populations and those covered by Medicare.

### Patient Sampling

All patients aged between 18 and 85 years with a T2D diagnosis, HbA_1c_ ≥8.0%, and practice portal account were sent an email to participate by their primary care clinician. Identification was automated in the practices’ EHR, and the email was sent 2 days after a laboratory result with an elevated HbA_1c_ level. The automated email, addressed by the primary care clinician, asked the patient to log in to the portal, which alerted the patient that their diabetes seemed poorly controlled. The system randomized patients in a 1:1 manner to receive either DEAP (intervention) or 1 page of information about diabetes (usual care control). No blinding or allocation concealment was used in this study.

### Intervention and Control Conditions

DEAP was integrated into the practices’ EHR, patient portal, and data warehouse. DEAP consisted of 9 self-directed DSMES modules for patients and care team alerts for clinicians to assist patients requesting additional help. The DEAP modules covered the *Standard 6: Curriculum* from the *National Standards for Diabetes Self-Management Education and Support* [14]. The 9 modules included: (1) diabetes disease process and general treatment, (2) nutritional management, (3) physical activity, (4) medications, (5) monitoring blood glucose, (6) acute complications, (7) chronic complications, (8) mental health, and (9) goal setting. Patients were sent modules in order and received biweekly reminders until they completed the modules. The next module was sent when a patient completed a module or after 7 days of noncompletion, which allowed patients to skip or ignore the modules*.*

Each module included 1 to 3 handouts and 1 to 3 videos for patients to review (Multimedia Appendix 1). Content was selected from existing publicly available and validated material from the ADA, National Diabetes Education Program, American Association of Diabetes Educators, Mayo Clinic, MedlinePlus, and other sources. Content was selected by the research team with support from 2 certified diabetic educators, a lay community educator, and 2 patients with T2D. Inclusion criteria for content consisted of being clear and understandable, evidence based, and engaging. Upon completion of a module, patients were asked 4 questions to assess their knowledge, 1 question to assess their confidence in managing the module’s domain, and 1 question to understand if the patient wanted additional help from the care team related to the content in the module. DEAP sent a summary of the patient’s responses to the primary clinician and provided an alert for patients reporting low confidence or requesting help in managing a domain.

Patients randomized to the usual care control group received 1 page of general diabetes information, which was equivalent to the handout information in the first DEAP module. They did not have access to the structured DEAP curriculum, knowledge or confidence assessments, or care team alerts.

### Measurements and Informatics

The patient portal and Privia electronic data warehouse were used to track patient progress through the curriculum, indicate whether modules were accessed and completed (completion was measured as a patient answering all postmodule questions), and record responses to end-module questions. The EHR was used to determine patient eligibility, measure patient characteristics (gender, age, race, ethnicity, preferred language, and insurance type), and capture health outcomes (HbA_1c_, BMI, and BP). Health outcomes for measuring effectiveness included HbA_1c_ (primary outcome) and BMI and BP (secondary outcomes), captured at baseline, 3 months, and 6 months. Implementation measures consisted of knowledge, confidence, adoption, and reach. Confidence was assessed using a Likert scale ranging from *not confident at all* to *completely confident*. *Adoption* was defined as the number of practices that were willing to participate in the study. We defined *reach* as the percentage of patients who agreed to participate in the study, the percentage of patients who started the DEAP curriculum within the intervention group, the percentage of patients who completed the DEAP curriculum, and the total number of DEAP modules that were accessed.

### Statistical Analysis and Sample Size Justification

We conducted both an intent-to-treat analysis of all intervention versus usual care control patients and a per-protocol analysis of intervention patients who completed at least one module (representing minimal intervention exposure) versus control patients. For both models, we made baseline-adjusted comparisons of 3- and 6-month means for HbA_1c_, BMI, and systolic BP (SBP) and diastolic BP (DBP) between the study groups. Using linear mixed models, health outcomes (HbA_1c_, BMI, and BP) at 3 and 6 months were modeled against a 2-level fixed group effect (intervention or control), the baseline value of that health outcome measurement, and a group-baseline interaction effect; the interaction term was removed if it was not significant at the 10% level and the Bayesian Information Criterion was lower in the no-interaction model. As an additional sensitivity analysis, unadjusted comparisons of the change in mean HbA_1c_, BMI, and BP over time and between the study groups were made using linear mixed models, including continuous health outcomes (HbA_1c_, BMI, and BP), a 2-level fixed group effect (intervention or control), a 3-level fixed time effect (baseline, 3 months, and 6 months), a fixed group-time interaction effect, and a patient-level random effect to account for within-participant dependence because of repeated measurements over time. The MEANS, FREQ, and GLIMMIX procedures in SAS statistical software (version 9.4 were used for analysis.

Sample size calculations were based on the assumption that 50% of participants would either decline to participate or not complete the study; therefore, recruiting 320 eligible participants would help ensure that 80 patients would participate and finish the study in each group (160 in total). Assuming a 5% type I error rate and an HbA_1c_ SD of 2 [4,15], we estimated over 80% power to declare mean HbA_1c_ for the intervention group to be significantly lower than in the usual care control group at either 3 or 6 months by at least 1 unit.

## Results

### Implementation Analyses

#### Adoption

The original plan was to recruit 4 practices from Privia’s network. However, we encountered significant practice enthusiasm across the organization, and a total of 21 practices across 5 states participated in the study. After the study was completed, Privia’s network extended DEAP to all practices as part of their standard operations.

#### Reach

The frequencies and percentages of intervention patients who accessed each of the training modules (and the numbers and percentages of those patients answering at least one question in each module and completing each module) are reported in Tables 1 and 2. Of the 189 intervention patients accessing at least the first module, the vast majority (140/189, 74.1%) eventually accessed all 9 modules, whereas only a few (8/189, 4.2%) failed to continue. Between 14% (21/151) and 28% (54/189) of the patients starting each module answered at least one of the corresponding postmodule questions. Of the 63 patients who answered at least one question in any module, 53 (84%) completed the questions to at least one module, with the majority answering at least one question completing all questions in each module.

**Table 1:** Intervention patients (n=189) who accessed, started, and completed particular Diabetes Engagement and Activation Platform modules.

Module	Accessed (n=189), n (%)^a^	Started^b^		Completed^c^	
		Total participants, n	n (%)	Total participants, n	n (%)
1. Basic assessment	189 (100.0)	189	54 (28.6)	54	34 (62.9)
2. Nutrition	181 (95.8)	181	34 (18.7)	34	33 (97.0)
3. Exercise	173 (91.5)	173	36 (20.8)	36	32 (88.8)
4. Mediations	167 (88.4)	167	25 (15.0)	25	23 (92.0)
5. Blood sugar	160 (84.6)	160	25 (15.6)	25	23 (92.0)
6. Acute complications	154 (81.4)	154	25 (16.2)	25	23 (92.0)
7. Chronic diabetes	151 (79.8)	151	21 (13.9)	21	21 (100.0)
8. Mood	146 (77.2)	146	22 (15.1)	22	17 (77.2)
9. Healthy goals	140 (74.1)	140	20 (14.3)	20	15 (75.0)

^a^Percentage calculated as 100 × (frequency accessed/189)%.

^b^Percentage calculated as 100 × (frequency started/frequency accessed)%.

^c^Percentage calculated as 100 × (frequency completed/frequency started)%.

**Table 2:** Number of Diabetes Engagement and Activation Platform modules accessed, started, and completed by intervention patients (n=189).

Number of modules accessed, n	Accessed, n (%)^a^	Started, n (%)^b^	Completed, n (%)^c^
0	N/A^d^	126 (66.6)	136 (71.9)
1	8 (4.2)	24 (12.6)	16 (8.4)
2	8 (4.2)	5 (2.6)	7 (3.7)
3	6 (3.1)	7 (3.7)	6 (3.1)
4	7 (3.7)	3 (1.5)	2 (1.0)
5	6 (3.1)	2 (1.0)	2 (1.0)
6	3 (1.5)	1 (0)	4 (2.1)
7	5 (2.6)	4 (2.1)	2 (1.0)
8	6 (3.1)	2 (1.0)	9 (4.7)
9	140 (74.0)	15 (7.9)	5 (2.6)

^a^Percentage calculated as 100 × (frequency accessed/189)%; mean 7.7, SD 2.5.

^b^Percentage calculated as 100 × (frequency started/189)%; mean 1.4, SD 2.7.

^c^Percentage calculated as 100 × (frequency completed/189)%; mean 1.2, SD 2.5.

^d^N/A: not applicable.

#### Patient Knowledge, Confidence, and Help Seeking

Patients answered a majority of knowledge questions correctly for each module (Table 3). The 4 most commonly missed questions included understanding what the HbA_1c_ measured, causes of low blood sugar, recommended number of daily servings of fruits and vegetables, and strategies for reducing cardiovascular risk. Upon completion of a module, most patients reported being very or completely confident of the module’s content. Only 18 patients asked for additional help from the care team after completing a module, most commonly after completing the introduction module (9/54, 17%), nutrition module (4/33, 12%), and exercise module (2/35, 6%).

**Table 3:** Summaries of knowledge assessment, confidence question, and desire to be contacted for each Diabetes Engagement and Activation Platform module.

Module	Correct knowledge questions			Confidence question			Expressed desire to be contacted	
	Sample size, n^a^	Mean (SD)	Not or a little confident, n (%)		Somewhat, very, or completely confident, n (%)	Sample size, n		Participants, n (%)
1. Basic assessment	34	3.6 (0.54)	12 (24)		37 (76)	54		9 (17)
2. Nutrition	33	2.9 (0.77)	16 (47)		18 (53)	33		4 (12)
3. Exercise	32	3.7 (0.52)	16 (47)		18 (53)	35		2 (6)
4. Mediations	23	3.7 (0.54)	2 (8)		23 (92)	24		0 (0)
5. Blood sugar	23	3.7 (0.65)	8 (33)		16 (67)	24		1 (4)
6. Acute complications	23	3.3 (0.88)	7 (28)		18 (72)	24		0 (0)
7. Chronic complications	21	3.0 (0.38)	6 (29)		15 (71)	18		0 (0)
8. Mood	17	3.7 (0.77)	9 (43)		12 (57)	22		1 (5)
9. Healthy goals	15	3.9 (0.26)	4 (21)		15 (79)	19		1 (5)
All modules	5	31.8 (2.17)	N/A^b^		N/A	N/A		N/A

^a^Sample sizes for each column can be different.

^b^N/A: not applicable.

### Effectiveness Analyses

**Figure 1:**
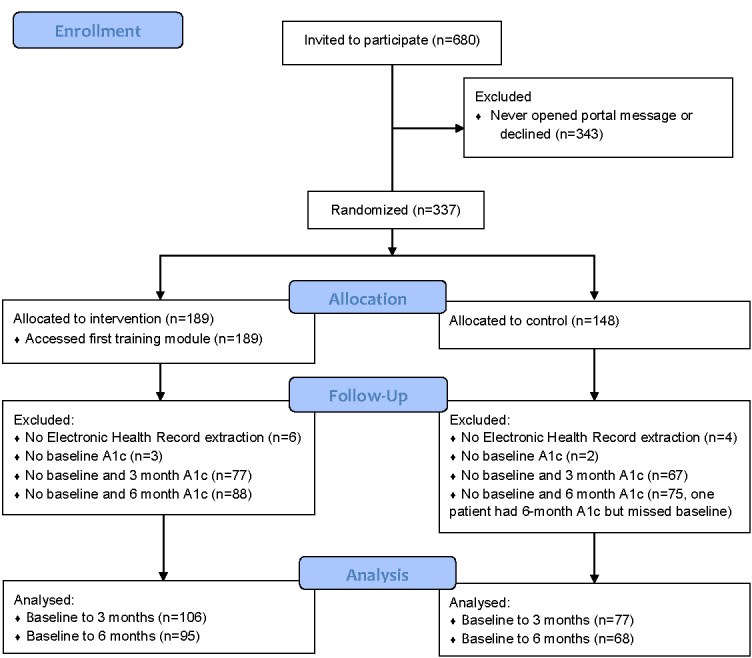
CONSORT (Consolidated Standards of Reporting Trials) flow diagram.

A total of 680 patients met the eligibility criteria and were emailed the portal invitation (Figure 1). Of those, 343 either never opened the portal message or after opening the message decided not to proceed with participation. Of the remaining 337 patients, 189 were randomly allocated to the intervention group and 148 to the control group. We identified 327 of the allocated patients in the EHR group (183 patients in the intervention group and 144 patients in the control group). All intervention patients (100%) accessed the first training module, with a percentage decrease for each successive module, and 74% (140/189) accessed the ninth module. Between 14% (21/151) and 28% (54/189) of the patients accessing the modules answered at least one of the corresponding postmodule questions, and 53 completed at least one module. A summary of patient characteristics and demographics are presented in Table 4. The average patient was just above 60 years, had an HbA_1c_ level >9, had a BMI in the obese range (>30), and had controlled BP (SBP<140). Both groups had similar rates of men and women, whereas the majority of participants were non-Hispanic, White, with English as their preferred language. Most participants had commercial health insurance or Medicare.

**Table 4:** Patient demographics at baseline.

Characteristics			Intervention				Control		
			Total participants, n		Value		Total participants, n		Value
Age (years), mean (SD)			183		61.1 (12.6)		144		60.6 (15.0)
HbA_1c_^a^, mean (SD)			180		9.3 (1.3)		142		9.6 (1.6)
BMI (kg/m^2^), mean (SD)			179		33.4 (7.0)		136		32.1 (7.1)
Systolic blood pressure (mm Hg), mean (SD)			180		129.5 (13.7)		137		128.7 (16.3)
Diastolic blood pressure (mm Hg), mean (SD)			180		76.7 (9.3)		136		77.8 (10.9)
**Sex, n (%)^b^**									
	Female	183		75 (40.9)		143		64 (44.7)	
	Male	183		108 (59.0)		143		79 (55.2)	
**Race, n (%)**									
	Asian	155		14 (9.0)		113		15 (13.3)	
	Black	155		16 (10.3)		113		13 (11.5)	
	Other	155		12 (7.7)		113		15 (13.3)	
	White	155		113 (72.9)		113		70 (61.9)	
**Ethnicity, n (%)**									
	Hispanic	137		3 (2.2)		97		9 (9)	
	Non-Hispanic	137		134 (97.8)		97		88 (90.7)	
**Language, n (%)**									
	Non-English	176		3 (1.7)		136		3 (2.2)	
	English	176		173 (98.3)		136		133 (97.8)	
**Insurance type, n (%)**									
	Medicaid	183		1 (0.5)		143		0 (0.0)	
	Medicaid	183		47 (25.7)		143		37 (25.8)	
	None	183		6 (3.2)		143		1 (0.6)	
	Commercial	183		127 (69.4)		143		105 (73.4)	
	Unknown	183		2 (1.1)		143		0 (0.0)	

^a^HbA_1c_: glycated hemoglobin.

^b^Percentage of sample with an event.

#### Intent-to-Treat Analysis

Table 5 contains summaries of the comparisons of mean health outcomes between intervention and control groups. There was no evidence that the mean for the primary outcome (HbA_1c_) was lower in the intervention group than in the control group at 3 months (8.0% vs 8.2%; *P*=.38) or at 6 months (8.2% vs 8.4%; *P*=.27). The mean BMI was significantly reduced in intervention group patients relative to control group patients at 3 months (31.7 kg/m^2^ vs 32.1 kg/m^2^; *P*=.04) and at 6 months (32.5 kg/m^2^ vs 33.0 kg/m^2^; *P*=.02). There was no evidence of improved SBP or DBP in the intervention group patients compared with the controls. Results were similar in the changes comparison analyses (Table 6), with no evidence of differences in baseline and 3-month changes between groups for any measures, and with only the change in BMI between baseline and 6 months for intervention group patients (–0.4 kg/m^2^ decrease vs 0.1 kg/m^2^ increase; *P*=.02).

**Table 5:** Comparisons of baseline-adjusted health outcome means between groups at 3 and 6 months.

Groups			Intervention									Control		
			Completed ≥1 module				All intervention							
			n		Mean^a^ (95% CI)		n		Mean (95% CI)		n			Mean (95% CI)
**HbA_1c_^b^**														
	3 months^c^	36		7.6 (7.2 to 8.0)		106		8.0 (7.7 to 8.4)		77			8.2 (8.0 to 8.6)	
	6 months^d^	25		7.9 (7.3 to 8.5)		95		8.2 (7.8 to 8.6)		69			8.4 (8.1 to 8.9)	
**BMI**														
	3 months^e^	40		31.3 (30.9 to 31.7)		138		31.7 (31.5 to 32.0)		100			32.1 (31.8 to 32.4)	
	6 months^f^	33		31.6 (31.1 to 32.0)		120		32.5 (32.2 to 32.8)		81			33.0 (32.7 to 33.4)	
**SBP^g^**														
	3 months^h^	40		124.0 (119.3 to 128.6)		136		126.2 (123.4 to 129.1)		105			126.9 (124.0 to 129.9)	
	6 months^i^	32		126.2 (121.7 to 130.8)		122		127.4 (124.6 to 130.2)		83			127.6 (124.5 to 130.7)	
**DBP^j^**														
	3 months^k^	40		72.3 (69.5 to 75.0)		136		74.9 (73.1 to 76.6)		105			75.9 (74.1 to 77.8)	
	6 months^l^	32		74.0 (71.0 to 77.0)		122		75.0 (73.0 to 77.0)		83			75.4 (73.2 to 77.6)	

^a^Mean: baseline-adjusted sample predicted value.

^b^HbA_1c_: glycated hemoglobin.

^c^Intent-to-treat (ITT) analysis (comparison between intervention and control patients; control-intervention): difference=0.2, 95% CI –0.2 to 0.6; *P*=.38 (indicates the interaction term left in the model). Per-protocol (PP) analysis: comparison between intervention subjects completing at least one Diabetes Engagement and Activation Platform module (answering postmodule questions) and control patients. PP analysis (control-intervention): difference=0.6, 95% CI 0.1 to 1.1; *P*=.03.

^d^ITT analysis (control-intervention): difference=0.3, 95% CI –0.2 to 0.8; *P*=.27. PP analysis (control-intervention): difference=0.5, 95% CI –0.2 to 1.2; *P*=.17.

^e^ITT analysis (control-intervention): difference=0.4, 95% CI 0.0 to 0.8; *P*=.04 (indicates the interaction term left in the model). PP analysis (control-intervention): difference=1.0, 95% CI 0.5 to 1.4; *P*<.001.

^f^ITT analysis (control-intervention): difference=0.5, 95% CI 0.1 to 1.0; *P*=.02. PP analysis (control-intervention): difference=1.0, 95% CI 0.5 to 1.5; *P*<.001.

^g^SBP: systolic blood pressure.

^h^ITT analysis (control-intervention): difference=0.7, 95% CI –3.4 to 4.9; *P*=.73. PP analysis (control-intervention): difference=3.2, 95% CI –2.3 to 8.8; *P*=.25.

^i^ITT analysis (control-intervention): difference=0.2, 95% CI –4.0 to 4.3; *P*=.94. PP analysis (control-intervention): difference=0.5, 95% CI –4.9 to 5.9; *P*=.85.

^j^DBP: diastolic blood pressure.

^k^ITT analysis (control-intervention): difference=1.1, 95% CI –1.4 to 3.6; *P*=.39. PP analysis (control-intervention): difference=4.3, 95% CI 1.0 to 7.5; *P*=.01.

^l^ITT analysis (control-intervention): difference=0.4, 95% CI –2.5 to 3.4; *P*=.78. PP analysis (control-intervention): difference=1.6, 95% CI –1.9 to 5.1; *P*=.37.

**Table 6:** Comparison between groups of change in glycated hemoglobin, BMI, and blood pressure from baseline to 3 and 6 months.

Groups		Intervention							Control	
		Completed ≥1 module				All interventions				
		n		Mean^a^ (95% CI)	n		Mean (95% CI)	n		Mean (95% CI)
**HbA_1c_^b^**										
	Baseline to 3 months^c^		36	–1.8 (–2.4 to –1.3)	106		–1.3 (–1.6 to –1.0)	77		–1.5 (–1.8 to –1.1)
	Baseline to 6 months^d^		25	–1.5 (–2.2 to –0.8)	95		–1.1 (–1.5 to –0.8)	68		–1.3 (–1.7 to –0.8)
**BMI**										
	Baseline to 3 months^e^		40	–0.9 (–1.3 to –0.6**)**	138		–0.3 (–0.5 to 0.0)	97		0.1 (–0.2 to 0.3)
	Baseline to 6 months^f^		33	–0.8 (–1.3 to –0.4)	119		–0.4 (–0.6 to –0.1)	78		0.1 (–0.1 to 0.4)
**SBP^g^**										
	Baseline to 3 months^h^		40	–5.0 (–10.2 to 0.2)	135		–3.8 (–6.5 to –1.2)	101		–1.7 (–4.9 to 1.5)
	Baseline to 6 months^i^		32	–1.7 (–6.7 to 3.4)	120		–0.4 (–2.9 to 2.1)	79		–1.1 (–4.2 to 2.1)
**DBP^j^**										
	Baseline to 3 months^k^		40	–5.2 (–8.1 to –2.2)	135		–2.4 (–4.0 to –0.8)	101		–1.3 (–3.1 to 0.6)
	Baseline to 6 months^l^		32	–2.6 (–5.9 to 0.8)	120		–0.4 (–2.2 to 1.4)	79		–1.3 (–3.4 to 0.8)

^a^Mean is the model-predicted difference (baseline minus the 3- or 6-month value).

^b^HbA_1c_: glycated hemoglobin.

^c^Intent-to-treat (ITT) analysis (control-intervention): difference=–0.2, 95% CI –0.6 to 0.3; *P*=.53 (comparison between all intervention and control patients). Per-protocol (PP) analysis (control-intervention): difference=0.3, 95% CI –0.3 to 1.0; *P*=.29 (comparison between intervention subjects completing at least one DEAP module [answering postmodule questions] and control patients).

^d^ITT analysis (control-intervention): difference=–0.1, 95% CI –0.7 to 0.4; *P*=.67. PP analysis (control-intervention): difference=0.2, 95% CI –0.6 to 1.1; *P*=.54.

^e^ITT analysis (control-intervention): difference=0.3, 95% CI 0.0 to 0.7; *P*=.07. PP analysis (control-intervention): difference=1.0, 95% CI 0.5 to 1.4; *P*<.001.

^f^ITT analysis (control-intervention): difference=0.5, 95% CI 0.1 to 0.9; *P*=.02. PP analysis (control-intervention): difference=1.0, 95% CI 0.5 to 1.5; *P*<.001.

^g^SBP: systolic blood pressure.

^h^ITT analysis (control-intervention): difference=2.1, 95% CI –1.9 to 6.2; *P*=.30. PP analysis (control-intervention): difference=3.3, 95% CI –2.8 to 9.4; *P*=.28.

^i^ITT analysis (control-intervention): difference=–0.7, 95% CI –4.6 to 3.2; *P*=.73. PP analysis (control-intervention): difference=0.6, 95% CI –5.3 to 6.5; *P*=.85.

^j^DBP: diastolic blood pressure.

^k^ITT analysis (control-intervention): difference=1.1, 95% CI –1.3 to 3.6; *P*=.35. PP analysis (control-intervention): difference=3.9, 95% CI 0.4 to 7.4; *P*=.03.

^l^ITT analysis (control-intervention): difference=–1.0, 95% CI –3.8 to 1.8; *P*=.47. PP analysis (control-intervention): difference=1.3, 95% CI –2.7 to 5.3; *P*=.52.

#### Per-Protocol Analyses

Comparisons among intervention group patients completing at least one DEAP module and controls are also provided in Table 5. Those who completed at least one module had a lower mean HbA_1c_ at 3 months compared with controls (7.6% vs 8.2%; *P*=.03), whereas there was no significant difference at 6 months (7.9% vs 8.4%; *P*=.17). Completers had significantly lower mean BMI at 3 months than controls (31.3 kg/m^2^ vs 32.1 kg/m^2^; *P*<.001) and at 6 months (31.6 kg/m^2^ vs 33.0 kg/m^2^; *P*<.001). There were no differences in SBP between completers and controls at 3 months (*P*=.25) and 6 months (*P*=.85). The intervention patients completing at least one module also had a larger mean DBP at 3 months than controls (72.3 mm Hg vs 75.9 mm Hg; *P*=.01), although there was no significant difference at 6 months (*P*=.37). Results from the comparison of change analyses (Table 6) were nearly identical, with the exception being that there was no evidence of different changes between groups in HbA_1c_ at 3 months (*P*=.29) or 6 months (*P*=.54). The change in BMI was significantly larger in those who completed at least one module compared with controls between baseline and 3 months (–0.9 kg/m^2^ vs 0.1 kg/m^2^; *P*<.01) and 6 months (–0.8 kg/m^2^ vs 0.1 kg/m^2^; *P*<.01), and with the change in DBP significantly larger in those intervention group patients completing at least one module than in controls (–5.2 mm Hg vs –1.3 mm Hg; *P*=.03).

## Discussion

### Principal Findings

DEAP uses publicly available material in a systematic manner to automatically provide virtual diabetes education and support through pre-existing patient portals. DEAP *Adoption* exceeded what was expected to meet the study objectives, indicating that clinicians recognize the need for innovative, structured, accessible DSMES to optimize patient care and outcomes. With regard to *reach*, more patients accessed and used DEAP modules (74%) and then would access other simple educational messages sent to patients (about 20% of general Privia educational messages were opened by patients). This uptake of the automated DEAP content is similar to that of traditional in person DSMES classes [16]. DEAP facilitated high levels of confidence, knowledge, and help-seeking behaviors.

Although knowledge does not always correlate with improved self-management [17], the DEAP intervention group demonstrated improved BMI relative to controls, whereas our per-protocol analysis also showed evidence of improvement in HbA_1c_ and DBP at 3 months postintervention for those completing modules. The lack of change in HbA_1c_ and BP may be because of dilution from non-DEAP users, who did not change. Nonetheless, the improved BMI in the intent-to-treat analysis is particularly impressive, given that most interventions to help patients lose weight must be fairly intensive, often including 25 or more hours of contact over 6 months [18].

DEAP leverages the existing use of patient portals [19] and compiles existing patient educational materials and videos into an easily accessible and understandable format. A key element of DEAP’s success is the automatic identification of patients with elevated HbA_1c_ within 2 days of the abnormal result, which removes the burden of identifying and engaging patients from the clinician and engages patients when they may be more amenable to making self-management changes. Another key element is that DEAP assembles publicly available information into a defined curriculum, making the material more acceptable and accessible to patients. Integrating DEAP into the clinician’s portal also comes with the imprimatur and credibility of the patient’s personal clinician.

Although we did observe benefits in this study comparing, we suspect that the benefits could have been greater if the automated self-directed learning was better coupled with support from the care team. How clinicians and care team members addressed the alerts was left to their discretion. Future implementations of DEAP could focus on alerting specific care team members when patients completed modules that could contact patients and offer additional ancillary services. For example, DEAP could notify a nutritionist when a patient expressed low confidence in managing their diet or missed a knowledge question [20] or a pharmacist about their medication management [21].

### Limitations

A limitation of this study is the short time frame, as 6 months of follow-up may not be enough for DSMES to lead to substantial and sustainable behavioral or health changes. However, the shorter time frame resulted in a greater improvement in BMI observed in the intervention group compared with the control and the improved HbA_1c_, BMI, and DBP observed among DEAP users compared with nonusers. The generalization of these results may be limited by the predominantly White, English-speaking, and non-Hispanic study sample, although the use of multiple practices and the focus on patients seen in primary care are strengths. Another factor limiting generalization was requiring a patient portal account for inclusion; investigations of approaches to encourage portal uptake or delivery of DEAP through other mechanisms are warranted.

### Conclusions

This low-intensity intervention to provide virtual diabetes self-management education proved both feasible and effective. The model is scalable, builds on existing infrastructures in many practices and health systems, and can be extended to other settings or conditions. Studying how automated self-directed approaches could be better linked with alerting care team members for additional directed care could have even greater benefits.

## Appendix

Multimedia Appendix 1 Diabetes Engagement and Activation Platform diabetes self-management education and support curriculum with sample content.

Multimedia Appendix 2 CONSORT Checklist.

## Abbreviations

ADA: American Diabetes Association
BP: blood pressure
DBP: diastolic blood pressure
DEAP: Diabetes Engagement and Activation Platform
DSMES: diabetes self-management education and support
EHR: electronic health record
HbA_1c_: glycated hemoglobin
PHR: personal health record
RCT: randomized controlled trial
SBP: systolic blood pressure
T2D: type 2 diabetes
