# Supplementary material for: Diabetes Engagement and Activation Platform for Implementation and Effectiveness of Automated Virtual Type 2 Diabetes Self-Management Education: Randomized Controlled Trial
Source: JMIR Diabetes. 2021 Mar 29;6(1):e26621. doi: 10.2196/26621 (PMC8088852; doi:10.2196/26621)
Supplement: Multimedia Appendix 1 [file diabetes_v6i1e26621_app1.docx]

| **Table S1. DEAP DSMES CURRICULUM WITH SAMPLE CONTENT** | |
| --- | --- |
| ***Diabetes Core Curriculum**** | **MedlinePlus Health Topics** |
| **Module 1:** Describing the diabetes disease process and treatment options | - **Title**: What is Diabetes and What Can I Do To Manage It? - **Basic Information:** Diabetes and Management Overview, Diabetes ABCs - **Videos:** Diabetes Overview***;*** Diabetes Treatment Overview |
| **Module 2:** Incorporating nutritional management into lifestyle | - **Title:** Eating Healthy to Self-Manage Type 2 Diabetes - **Basic Information:** Type 2 Diabetes Mellitus and Diet - **Videos:** Diabetes and Diet; Meal Planning; Plate Method; Healthy Eating at Family Gatherings and Social Events |
| **Module 3:** Incorporating physical activity into lifestyle | - **Title:** Activity and Exercise to Self-Manage Type 2 Diabetes - **Basic Information:** What I need to Know about Physical Activity and Diet; Exercise and Type 2 Diabetes; Stretching and Flexibility; - **Videos:** Physical Activity and Exercise: Practical Tips and Action Steps |
| **Module 4:** Using medication(s) safely and for maximum therapeutic effectiveness | - **Title:** Understanding and Managing Diabetes Medications - **Basic Information:** Oral Diabetes Drugs; Treatment with Insulin - **Video:** Taking Medications-Oral and Insulin; Missed Your Medication |
| **Module 5:** Monitoring blood glucose and using the results for self-management | - **Title:** Blood Glucose Monitoring and Decision Making - **Basic Information:** Blood Glucose Monitoring Overview, Skills, A1C Test - **Video:** Self-Blood Glucose Monitoring in Diabetes Mellitus Monitoring; Hyperglycemia (High Blood Sugar) and Hypoglycemia (Low Blood Sugar) |
| **Module 6:** Preventing, detecting, and treating acute complications | - **Title:** Preventing, Recognizing and Treating Illness - **Basic Information:** Vaccinations; Flu and Diabetes; Preventing Infection; Foot Care; Sick Day Plan; When to Seek Help - **Video:** Preventing Flu; 7 Diabetes Friendly Foods to Fight Colds and Flu |
| **Module 7:** Preventing, detecting, and treating chronic complications | - **Title:** Preventing, Recognizing and Treating Chronic Illness - **Basic Information:** Heart Health; Eye Health; Kidney Health; Neuropathy - **Videos:** Type 2 Diabetes and Blood Pressure; Foot Care |
| **Module 8:** Developing personal strategies to address psychosocial issues and concerns | - **Title:** Coping with Type 2 Diabetes - **Basic Information:** Depression and Diabetes; Stress Management; - **Videos:** Healthy Coping; Guided Imagery; Guided Meditation for Stress Management |
| **Module 9:** Personal strategies to promote health and behavior change | - **Title:** Success with Behavior Changes to Improve Your Health - **Basic Information:** Goal Setting; Strategies for Starting and Maintaining Healthy Behaviors; Changing Your Habits: Steps to Better Sleep; Quitting Smoking - **Videos:** Creating and Sustaining Behavior Change; Quit Smoking; Guided Meditation to Help Stop Smoking |
| *Standards Revision Task Force. National Standards for Diabetes Self-Management Education and Support. *Diabetes Care*. 37 Supplement 1, January 2014. | |
